# Mapping the Druggable Allosteric Space of G-Protein Coupled Receptors: a Fragment-Based Molecular Dynamics Approach

**DOI:** 10.1111/j.1747-0285.2010.01012.x

**Published:** 2010-09

**Authors:** Anthony Ivetac, J Andrew McCammon

**Affiliations:** 1 Department of Chemistry and Biochemistry, University of California San DiegoLa Jolla, CA 92093, USA; 2 Center for Theoretical Biological Physics, University of California San DiegoLa Jolla, CA 92093, USA; 3 Howard Hughes Medical Institute, University of California San DiegoLa Jolla, CA 92093, USA; 4 Department of Pharmacology, University of California San DiegoLa Jolla, CA 92093, USA

**Keywords:** allosteric, docking, fragment-based, GPCR, molecular dynamics

## Abstract

To address the problem of specificity in G-protein coupled receptor (GPCR) drug discovery, there has been tremendous recent interest in allosteric drugs that bind at sites topographically distinct from the orthosteric site. Unfortunately, structure-based drug design of allosteric GPCR ligands has been frustrated by the paucity of structural data for allosteric binding sites, making a strong case for predictive computational methods. In this work, we map the surfaces of the β_1_ (β_1_AR) and β_2_ (β_2_AR) adrenergic receptor structures to detect a series of five potentially druggable allosteric sites. We employ the FTMAP algorithm to identify ‘hot spots’ with affinity for a variety of organic probe molecules corresponding to drug fragments. Our work is distinguished by an ensemble-based approach, whereby we map diverse receptor conformations taken from molecular dynamics (MD) simulations totaling approximately 0.5 μs. Our results reveal distinct pockets formed at both solvent-exposed and lipid-exposed cavities, which we interpret in light of experimental data and which may constitute novel targets for GPCR drug discovery. This mapping data can now serve to drive a combination of fragment-based and virtual screening approaches for the discovery of small molecules that bind at these sites and which may offer highly selective therapies.

G-protein coupled receptors (GPCRs) represent a large and diverse superfamily of integral membrane proteins, triggering a wide range of signal transduction pathways in response to such stimuli as neurotransmitters, hormones and photons (1,2). Their ubiquity in the human genome and engagement in key physiological processes makes them a top class of pharmaceutical target (3). Testament to this, GPCRs are associated with approximately 30% of current drugs (4) and are linked to such diseases as cancer (5) and those of the cardiovascular and central nervous systems (6,7).

Despite encompassing over 1000 members, spread over six subfamilies (8), GPCRs share a common architecture consisting of seven transmembrane α-helices (TM1–TM7) connected by three intracellular loops (ICL1-ICL3) and three extracellular loops (ECL1-ECL3). Recent experimental evidence has demonstrated that GPCRs are highly dynamic structures, embracing a spectrum of conformational states from fully inactive through fully active (9). GPCR activity is manifested through their stimulation of G-proteins, which interact with the intracellular face of GPCRs and, in turn, engage with effector proteins to regulate levels of second-messenger molecules (10). Most GPCRs appear to possess an intrinsic, basal level of activity in the absence of any ligand. The binding of ligands (from the extracellular medium) then shifts the conformational equilibrium toward the fully active state in the case of agonists and toward the fully inactive state in the case of inverse agonists (11). To further emphasize the conformational heterogeneity of GPCRs, it has been established that ligands can regulate different downstream signalling cascades through the same GPCR. This phenomenon is known as ‘functional selectivity’ and is thought to reflect the ability of different ligands to stabilize highly specific receptor conformations (12).

G-protein coupled receptor structural biology has recently enjoyed a boom (13), with high-resolution crystallographic structures now available for the avian β_1_ adrenergic receptor (β_1_AR) (14), human β_2_ adrenergic receptor (β_2_AR) (15–17) and human adenosine A_2A_ receptor (18), as well as the proposed active conformation of bovine opsin in complex with a G-protein fragment (19). These structures have yielded great insights into the structure–function relationship between ligand binding and G-protein activation, whereby ligands are thought to stabilize/induce a variety of conformational rearrangements, which are characteristic of different states and can have diverse downstream effects (20–22). From a drug design perspective, the structures have afforded a detailed picture of the ligand-binding pocket, formed in an extracellular cleft leading to the transmembrane core, and have opened new drug discovery avenues (23). For example, new structure-based virtual screening efforts have emerged, in a traditionally ligand-based field, reporting the successful discovery of several new active compounds (24,25).

References to the GPCR ligand-binding site have, hitherto, alluded to the ‘orthosteric’ site, which is defined as the pocket bound by the endogenous activating ligand (26). GPCR drug discovery to date has predominantly been concerned with this site, yielding an impressive repertoire of drugs that compete with the endogenous ligand and generate a variety of efficacies. Such orthosteric ligands include well-known examples such as the anti-hypertension drug atenolol and the anti-asthma drug salbutamol. However, there has been formidable recent interest in compounds that modulate GPCR activity through an ‘allosteric’ mechanism [as reviewed in (26–29)]. Such allosteric ligands bind to a site that is topographically distinguished from the orthosteric site (OS) and thus do not compete with orthosteric ligands (30). Allosteric ligands may (i) modify the binding and/or efficacy properties of an orthosteric ligand (termed ‘allosteric modulators’) or (ii) affect the activation state of the GPCR by themselves (termed ‘allosteric agonists’) (31). The binding of an allosteric ligand may therefore be considered to stabilize/induce GPCR conformations that either increase/decrease the affinity of orthosteric ligand binding and/or affect G-protein stimulation. The main allure of allosteric ligands is their potential for greater receptor selectivity, by binding to specific GPCR subtypes (26). The strong evolutionary conservation of the OS (across closely related GPCRs) has caused problems with cross-reactivity of orthosteric ligands, which can lead to undesirable therapeutic side-effects. For example, orthosteric drugs that act on the β_1_AR for the treatment of heart disease may cross-react with the β_2_AR and cause unwanted pulmonary effects (and vice-versa for anti-asthma drugs that can cause cardiac effects) (32). Similarly, the development of orthosteric antipsychotic drugs, acting at the muscarinic acetylcholine receptor, has been plagued with a lack of subtype specificity, leading to side-effects and motivating the discovery of more selective allosteric drugs (33). The amino acid sequences of allosteric binding sites are more likely to have diverged among members of a receptor subclass than the OS, therefore conferring specificity and reducing the potential for off-target activity. A key advantage of allosteric modulators is that they exert no effect by themselves, serving only to tune the effect of endogenous ligands and thus causing less disruption to the normal physiological profile of the GPCR. This activity also has implications for toxicity, whereby, because the effect of allosteric modulators is saturable, overdose-associated risks are reduced. Finally, it has been noted that allosteric ligands identified to date display greater structural diversity than their orthosteric counterparts, thus opening up their chemical space and making them more amenable to modifications to solve ADMET problems (34). In summary, allosteric GPCR ligands have generated considerable excitement in the field of GPCR drug discovery and offer various modes of receptor modulation, with a degree of specificity unattainable at the OS.

Allosteric GPCR ligands have now been described for a wide range of GPCRs, including members from all three human classes (26,27,29). Two allosteric drugs have already been FDA-approved: cinacalcet, which binds the calcium-sensing receptor in hyperparathyroidism, and maraviroc, which binds the CCR5 chemokine receptor in HIV infection, as well as several candidates undergoing clinical studies (35). Perhaps the two best studied subclasses are those of the muscarinic receptors (Class A) and the metabotropic glutamate receptors (Class C), which bind allosteric ligands with diverse pharmacological activities and play important roles in the diseases of the central nervous system (6). Despite encouraging progress in the discovery and functional characterization of allosteric GPCR ligands, the structural biology of their binding sites is still poorly understood. An outstanding question is where do they bind? Site-directed mutagenesis has been employed to give the approximate locations and identify interacting residues for several allosteric ligands and receptors (31). Such experimental mapping studies have been invaluable in providing the first details of interaction sites and exposing their diversity, occurring in both solvent-exposed and bilayer locations (6). However, given the time and labor-intensive nature of such methods, as well as the need for an existing allosteric ligand, there is a strong case for the implementation of faster, more predictive approaches with which to identify putative allosteric binding sites. Such tools are particularly germane in the case of GPCRs, which have proven especially difficult to crystallize and therefore do not enjoy the same degree of structural data which has supported allosteric drug design in soluble proteins, such as kinases (34). Establishing the topographical location of allosteric sites in GPCRs would drive the structure-based, *de novo* discovery of allosteric ligands and help elucidate the structural basis of their function.

The recent milestone of high-resolution structural data for ligand-activated GPCRs provides a role for computational methods in allosteric drug discovery. The motivation to uncover new drug binding sites is not a new one and has been fueled by the characterization of several recent sites which have therapeutic potential (36). Given the three-dimensional structure of a target protein, a number of algorithms have been developed to scan the entire protein surface for cavities, which are capable of binding small molecules and are potentially druggable [as reviewed in (37,38)]. Such methods aim to detect and score such pockets based on various concepts of molecular recognition. These range from a purely geometric treatment of the binding pocket [e.g. PocketPicker (39)] to more rigorous energy-based calculations that typically attempt to dock a series of probe molecules to candidate pockets and estimate the strength of their interaction [e.g. GRID (40)]. FTMAP (41) is one of the most recent energy-based mapping algorithms and was originally conceived as a faster, computational equivalent of an experimental technique known as the multiple solvent crystal structures (MSCS) method (42). With the MSCS approach, the target protein is co-crystallized in the presence of diverse organic solvent probe molecules, and it has been demonstrated that the probes tend to cluster at functionally important sites. Similarly, FTMAP docks a panel of 16 probe molecules (representing a variety of functional groups and drug fragments) to the protein surface and uses an empirical scoring function to determine low-energy poses. FTMAP is distinguished from other methods by a combination of its clustering scheme, which differentiates between consensus sites (CSs) (which represent putative binding sites) and isolated, non-specific binding events and an efficient sampling method (41). FTMAP (and its predecessor CS-Map) have been validated against a range of pharmaceutical targets (including renin aspartic protease, elastase and glucocerebrosidase), showing excellent agreement with binding sites identified by X-ray crystallography, for both organic solvents and drug molecules (41,43,44). These encouraging correlations with existing structural data suggest that the FTMAP method also has the potential to work in a *prescriptive* fashion, in the identification of novel druggable allosteric binding sites.

The aforementioned mapping algorithms typically depend on the availability of one, or occasionally a few, experimentally determined atomic structures of the target protein. Considering the structural flexibility of proteins, this static representation of the target can be extremely restrictive and is a recognized flaw in many protein–ligand docking efforts (45,46). Consequently, a variety of schemes have been proposed that allow target flexibility to be taken into account and range from modeling simple sidechain changes to full backbone and sidechain mobility (47,48). In the context of binding site identification, the incorporation of protein flexibility is appealing as allosteric pockets may only form transiently and relatively infrequently in the dynamics of the protein and may therefore be missed in experimental structures. Also, the topography of allosteric sites may change, exposing different protein residues and altering their physicochemical properties. The role of flexibility is even more pronounced in the case of GPCRs, renowned for their strong intrinsic conformational plasticity that has hampered crystallization efforts (49). Molecular dynamics (MD) simulation is a popular method for the modeling of protein motions and generation of ensembles of protein conformations, which evolve from an experimental starting structure (50). Several MD simulation approaches have now been reported, examining the conformational dynamics of GPCRs, often with a view to capturing the structural rearrangements that accompany receptor activation [e.g. (51,52)]. MD simulations have also demonstrated their value in drug discovery applications by exposing dramatic binding site changes. For example, a relatively short (2 ns) MD simulation of HIV-1 integrase revealed a new inhibitor binding site that led to the discovery of the first integrase inhibitor, raltegravir (53). More subtle binding site dynamics have been used in virtual screening applications, whereby putative compounds are docked to a range of conformers, as opposed to a single, experimental structure, to improve rank ordering (54). It is therefore appealing to couple a computational mapping analysis with an MD-based ensemble of GPCR conformations, so as to potentially discover novel allosteric sites, as well as further characterizing any existing sites.

In this work, we report the computational mapping of potential allosteric sites on the surface of the human β_1_ (β_1_AR) and β_2_ (β_2_AR) adrenergic receptors (Figure 1), which may be exploited in the structure-based design of allosteric ligands. The recent milestone of high-resolution crystal structures for these pharmaceutically important GPCRs makes them ideally suited to such a mapping analysis. β_1_AR and β_2_AR are members of the β-adrenoceptor subfamily of Class A GPCRs and play key roles in heart muscle contraction and bronchial smooth muscle relaxation, respectively. They have therefore been targeted in the treatment of heart disease and pulmonary problems, through a range of orthosteric drugs. Unfortunately, these receptors typify a limitation endemic throughout GPCRs, and indeed many other drug targets, in the extremely high sequence conservation of their OSs (94% identity over 16 residues). Consequently, subtype selectivity is a key issue in their pharmacological control, and there is a pressing need to identify drugs with a greater capacity to discriminate between related receptors. Allosteric drugs pose one such solution, and we have therefore employed the FTMAP algorithm to search for druggable hot spots in non-orthosteric regions of the receptors. To address the role of conformational flexibility, we have applied FTMAP to both the experimental structure and an ensemble of 15 representative MD simulation structures, for each receptor. Here, we used the multicopy MD approach, whereby a series of six independent 40-ns trajectories was generated for each receptor in a phospholipid bilayer, yielding a combined total simulation time of approximately 0.5 μs. This study was inspired by previous work that used the CS-Map algorithm to expose novel binding sites in MD snapshots of a soluble viral target – the H5N1 influenza neuraminidase (55). The current work is distinguished by use of the improved FTMAP code and its application to a pair of human membrane protein targets, as well as a global search of the entire protein surface. Our results define a set of five key non-orthosteric regions that act as consensus binding sites for organic probes and which may represent targets for allosteric ligands. Interestingly, the sites are distributed across both solvent-exposed and lipid-exposed surfaces, and it appears that one site may be exclusive to one receptor subtype, while all others are generally shared. Furthermore, some of the sites are not apparent in the experimental structures, only being revealed in the MD-generated conformers. We characterize each site in the context of experimental data and propose they will serve to guide fragment-driven and virtual screening studies for the identification of allosteric compounds, which may be shared with related GPCRs.

**Figure 1:**
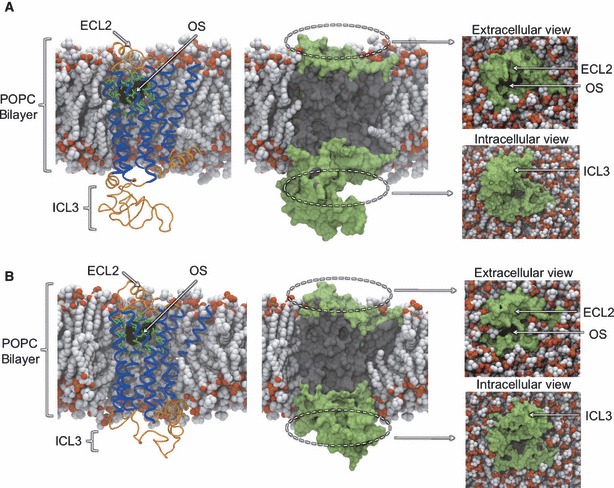
Snapshots of the bilayer-embedded β_1_AR (A) and β_2_AR (B) MD simulation systems. Receptors are shown in cartoon representation (left), with the seven TM helices colored blue and other structures colored orange. Residues comprising the orthosteric site (OS) are shown in green stick representation. The co-crystallized orthosteric ligand is superimposed and shown in black molecular surface representation. Receptors are shown in molecular surface representation (center, right) to illustrate the regions being mapped by probes. Solvent-accessible surfaces are colored green, while other regions are colored gray. Extracellular and intracellular views show the solvent-exposed regions in more detail, from above and below the bilayer. Palmitoyl-oleoyl phosphatidylcholine phospholipid molecules are shown in space-filling representation and are colored by atom type.

## Methods

### Mapping algorithm

Each receptor conformation was mapped for potential small molecule binding sites using the fragment-based FTMAP algorithm (41), through its online server (http://ftmap.bu.edu). FTMAP scans the global surface of the protein with a diverse library of 16 small organic probe molecules, with varying hydrophobicity and hydrogen-bonding capability (ethanol, isopropanol, isobutanol, acetone, acetaldehyde, dimethyl ether, cyclohexane, ethane, acetonitrile, urea, methylamine, phenol, benzaldehyde, benzene, acetamide and *N,N*-dimethylformamide). A fast Fourier transform correlation approach is used for this step, sampling billions of receptor–probe complexes. Each complex is evaluated using an energy expression that includes terms for the van der Waals and electrostatic interaction energy, as well as solvation effects. The 2000 most favorable docked positions of each probe are then energy-minimized and clustered. For each probe, the six clusters with the lowest mean interaction energy are retained. Next, these low-energy clusters from different probe types are themselves clustered into CSs, which define hot spots where multiple probes congregate with high affinity. It has been shown that such behavior is characteristic of a druggable site, which can therefore be distinguished from unimportant isolated sites. The CSs are ranked by the number of probe clusters they incorporate, with the largest CSs proposed to represent the most important sites of interest. In this work, the 10 largest CSs have been analyzed – named CS1 (largest CS) through CS10 (smallest CS). Note that multiple CSs may aggregate to form a single binding site, by representing the different moieties of a ligand. The main output from the FTMAP server is a Protein Data Bank (PDB) file containing the analyzed protein structure along with representative probe molecules belonging to the clusters forming the CSs. By definition, these hot spots typically contain a mixture of probe types, representing a variety of sizes and non-bonded interactions with the protein. The output also contains useful statistics on the specific protein residues that mediate non-bonded protein–probe interactions and therefore contribute to the binding energy.

### MD simulation setup

The 2.7 Å crystal structure of cyanopindolol-bound turkey β_1_AR [PDB code 2VT4 (14)] was used as a template to model human β_1_AR, with cyanopindolol removed from the OS. A pairwise sequence alignment of the turkey and human sequences was generated using MUSCLE (56), yielding a high sequence identity of 81%. The alignment was composed of two contiguous regions, separated by the deleted ICL3: an N-terminal region containing TM1-TM5 and a C-terminal region containing TM6-TM7. The MODELLER 9v4 package (57) was used to generate a set of homology models of human β_1_AR from the alignment, with the lowest energy conformation (based on the MODELLER energy term) selected for this study. An initial conformation of the absent ICL3 was modelled between TM5 and TM6. A short energy minimization of the model structure was carried out with the GROMACS package (58). The final β_1_AR structure represents residues 56 through 393.

The 2.4 Å crystal structure of carazolol-bound human β_2_AR [PDB code 2RH1 (15)] was used for human β_2_AR, with carazolol removed from the OS. The MODELLER 9v4 package was used to model an initial conformation of the absent ICL3 between TM5 and TM6. A short energy minimization of the model structure was carried out with the GROMACS package (58). The final β_2_AR structure represents residues 29 through 342.

To accurately model the position of the receptors in a phospholipid bilayer and allow relaxation of the modelled ICL3 in a membrane environment, a coarse-grained (CG) simulation step was used. CG simulations have been demonstrated as a rapid method for the self-assembly of protein–lipid complexes and are therefore useful in generating the initial co-ordinates for conventional MD simulations of membrane proteins [see http://sbcb.bioch.ox.ac.uk/cgdb & (59)]. For each receptor, the all-atom protein structure was first converted into a reduced CG representation and surrounded with 256 randomly positioned CG palmitoyl-oleoyl phosphatidylcholine (POPC) molecules. This system was then solvated with approximately 5400 CG water particles and counterions. After energy minimization, an 800-ns (effective time) CG simulation was performed, whereby the POPC lipids self-assembled into a bilayer phase around the inserted receptor. All non-ICL3 residues were distance-restrained, such that the core experimental structure was preserved, while the modelled ICL3 loop was allowed to move in the solvent. As described in (60), the 800-ns snapshot of the CG system was converted into atomistic detail, by first superimposing the atomistic receptor model onto the CG receptor model co-ordinates. The modelled ICL3 conformation was then replaced with that captured in the final CG snapshot, using the Pulchra algorithm (61). Next, the CG lipids were converted into atomistic detail using a library of 1500 atomistic POPC lipids taken from a simulation of a pure POPC bilayer. The atomistic lipid with the lowest root-mean-square deviation (RMSD) to the CG lipid backbone was used as a replacement. The 256-lipid reconstructed bilayer was enlarged to 359 lipids to avoid periodicity artifacts in the atomistic simulations, using molecules from periodic images. Finally, the protein–bilayer complex was solvated with approximately 30 000 water molecules and counterions to preserve electroneutrality. The co-crystallized ligands in the orthosteric binding site were thus replaced with water molecules.

The final simulation cell was of dimension 11 nm × 11 nm × 12 nm and contained approximately 113 000 atoms. The bilayer-inserted positions of both receptors and final conformations of ICL3 can be seen in Figure 1. A final energy minimization of the entire system was followed by a 3-ns equilibration phase, whereby all receptor heavy atoms were position-restrained to allow a relaxation of the packing of the lipids and solvent around the protein. Unrestrained production runs were then simulated for 40 ns each. A total of 6 × 40 ns trajectories were generated for each receptor, which were then concatenated into a single 240-ns trajectory for clustering. Such a multicopy technique has been shown to improve conformational sampling in conventional MD simulations, when compared to a single long simulation (62).

### MD simulation details

All ionizable protein residues were assigned default protonation states except for Glu-92 in β_1_AR and the equivalent Glu-94 in β_2_AR, which were neutralized as they adopt lipid-exposed conformations. The amino and carboxyl termini of the receptors were also neutralized as they do not correspond to the actual receptor termini. The initial CG simulation step was performed using the MARTINI forcefield (63) with the GROMACS v.3.3.1 MD simulation package (58,64,65) and a 20 fs timestep. For the conventional MD simulations, all energy minimization was performed using 100 steps of the steepest descents algorithm. Equilibration was performed using harmonic restraints on all protein heavy atoms (force constant = 1000 kJ/mol/nm^2^), a Berendsen thermostat (66) and a Berendsen barostat to maintain pressure at 1 bar (66). Convergence of the potential energy and volume of the system was used to ensure adequate lipid and solvent relaxation during equilibration. A separate equilibration (and subsequent production run) was performed for each of the six copies of each system using a different randomization seed for the initial atomic velocities (62). The unrestrained production runs were performed with a Nosé–Hoover thermostat (67,68), and pressure was maintained at 1 bar by a Parrinello–Rahman barostat (69). Simulations were run in the NPT ensemble and at a temperature of 310 K. Particle mesh Ewald was used to treat long-range electrostatics (70), and the single point charge water model (71) was used for the solvent (as recommended for the GROMOS force field). Chloride anions were positioned randomly among the solvent to neutralize the net positive charge of the protein. An integration time step of 2 fs was used. The LINCS algorithm was used to restrain all bond lengths (72). Simulations were set up, performed and analyzed using the GROMACS v.3.3.1 MD simulation package (58,64,65). The GROMOS96 force field was used (43A1 parameter set) (73), with lipid parameters based on those described in (74). All molecular graphics were produced with Visual Molecular Dynamics (VMD) (75).

### Selecting representative MD structures

An ensemble of 15 MD structures, representative of each 240-ns receptor trajectory, was generated using an RMSD-based conformational clustering algorithm. This approach has been shown to be an effective way of capturing the conformational diversity of an MD ensemble in a reduced dataset (76). For each 240-ns trajectory, simulation frames were saved every 50 ps, yielding a set of 4800 protein snapshots. All snapshots were superimposed using the Cα atoms of nine key secondary structures to remove overall rotation and translation: the seven TM helices, the 8th helix that lies on the intracellular face of the membrane and the short helix contained within ECL2 (representing 229 residues in β_1_AR and 227 residues in β_2_AR). The RMSD-based clustering step was carried out on the same set of Cα atoms, using the ‘g_cluster’ component of the GROMACS package and a 1.5-Å cutoff. This large set of residues was chosen as we are performing a global search of binding sites and are therefore interested in receptor-wide structural changes. The top 15 clusters of similar structures were retained, representing 96.8% of the β_1_AR trajectory and 99.4% of the β_2_AR trajectory. The centroid structure from each cluster was used as the cluster representative for mapping analysis (see Supporting Information for distribution of cluster centroids).

## Results and Discussion

### MD simulation dynamics

**Figure 2:**
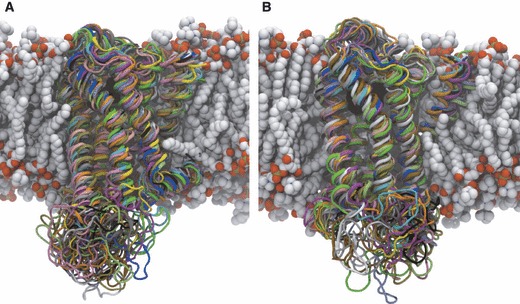
Snapshots of the ensemble of 15 representative structures extracted from β_1_AR (A) and β_2_AR (B) MD simulations. The aligned receptor structures are shown in cartoon representation, with the experimental conformation colored in blue and all 15 MD conformations colored independently. Palmitoyl-oleoyl phosphatidylcholine (POPC) phospholipid molecules are shown in space-filling representation and are colored by atom type. The structure of the POPC bilayer shown is taken from the beginning of the MD simulations.

Before discussing the mapping results, we briefly characterize the structural variation captured in the MD simulations. To assess the conformational drift of the structures, we measured the RMSD with respect to the experimental starting co-ordinates (see Supporting Information). Using the Cα atoms of the TM helices for an indication of global drift, the RMSD varies between 1.5 and 3 Å, typical of many membrane protein simulations and indicative of structures relaxing from their crystallization environment. The notable difference in the drift of the individual simulation copies is a hallmark of the multicopy approach and is suggestive of the greater variation in sampling compared to a single trajectory. It is also apparent that the structures of some copies are still evolving from the experimental structure, which may be attributed to the simulation of the ligand-free state, the inherent structural plasticity of GPCRs and timescale limitations. Figure 2 shows the superposition of the MD ensemble and the experimental structure to illustrate the substantial conformational changes that take place during the MD simulations. As expected, the large, solvent-exposed ICL3 shows the greatest movement, swinging toward and away from the TM core of the protein, and in the case of β_1_AR occasionally plugging the ‘intracellular mouth’ at the center of the TM helices. We observe significant variations in the shape and size of this solvent-exposed cavity, which has been shown to open to accommodate portions of the G-protein in an opsin crystal structure (19). On the extracellular surface, it has been predicted from experimental structures that ECL2 is constrained as it is tethered to the TM core through a disulfide bridge to TM3, as well as containing an internal disulfide bridge (21). While these bonds certainly restrict the conformational freedom of ECL2, we observe motions that, in concert with other extracellular features, modulate the accessibility of the OS by opening and closing the ‘extracellular mouth’. This gating function, seen in both receptors, may have important implications for the mechanism of allosteric modulation in this region and is discussed in more detail later. In the TM region of the protein, we observe inter-helical packing adjustments that may expose ligand-binding opportunities in the hydrophobic environment of the bilayer core. For example, TM1 exhibits strong fluctuations in its packing against the TM bundle, often losing interactions with TM2 and moving away from the core. Intra-helical changes are also apparent, for example the well-known proline-induced kinking of TM6, which is thought to be a conserved conformational switch in GPCR activation and may be important in permitting GPCR:G–protein interactions. In summary, a variety of structural rearrangements are embodied in the MD ensemble, therefore presenting diverse topographies more representative of GPCR dynamics than experimental structures alone.

### Mapping results

**Figure 3:**
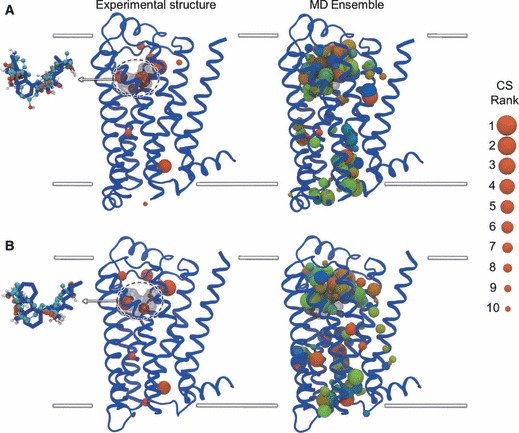
Mapping results for the β_1_AR (A) and β_2_AR (B) structures, showing the distribution of hot spots. The top 10 consensus sites (CSs) are shown as spheres, depicting the center-of-mass of the hot spot and the radius of which denotes the ranking of the CS (see legend). The mapping of the experimental structure is shown on the left, with the inset showing the probe molecules from the three CSs that overlap with the co-crystallized ligand in the orthosteric site. The mapping of the MD ensemble is shown on the right, with all 15 sets of CSs displayed simultaneously and colored on a RGB color scale from red (MD cluster 1) through blue (MD cluster 15). Receptors are shown in blue cartoon representation and depict the experimental conformation. The superimposed co-crystallized ligands are shown in transparent black molecular surface representation (main) and blue stick representation (inset). Inset probe molecules are shown in ball-and-stick representation, colored by atom type. Approximate limits of the bilayer are shown as white lines.

The results of the mapping of the experimental and MD structures of both receptors are summarized in Figure 3, by depicting the global distribution of the top 10 CSs identified. Before examining the allosteric hot spots, it is useful to assess the identification of the OS in the experimental structures, which were both solved in the presence of orthosteric ligands. In each receptor, three neighbouring CSs superimpose very well with fragments of the orthosteric ligand (Figure 3, inset), with their constituent probe molecules tracing the ‘L’-shaped geometry of the ligand and detecting ring moieties. For β_1_AR, these CSs are ranked 1st, 4th and 6th, while for β_2_AR they are ranked 2nd, 5th and 10th. This demonstrates that FTMAP is capable of finding low-energy probe sites that correspond to experimentally determined drug fragment sites and is consistent with applications to other targets that also detect high overlap between probes and drug molecules (41). The co-crystallized orthosteric drugs are relatively large for known βAR ligands and likely cover the majority of the OS. It is therefore reasonable to consider all other hot spots as potential allosteric binding sites (referred to as ‘non-orthosteric’). For the experimental structures, the non-orthosteric CSs are mainly found in subregions of the extracellular and intracellular mouths, with smaller CSs located in two lipid-exposed regions. The highest ranked non-OSs are both found on solvent-exposed surfaces and the overall distribution of sites is very similar for both receptors. The majority of CSs are contained within the bounds of the bilayer thickness with the exception of a single CS exposed to the intracellular bulk solvent in β_1_AR. Switching our focus to the mapping of the MD ensemble, it is clear again that the extracellular mouth is the richest source of CSs in both receptors, spanning both orthosteric and non-OSs and exploring new regions compared to the experimental structures. The intracellular mouth is also an important region for many probes, some of which penetrate more deeply into the TM core than in the experimental structures, almost to the center of the bilayer in the case of β_1_AR. Interestingly, novel sites are found in the lipid-exposed surfaces of the MD structures, which were not apparent in the experimental structures, forming two CS clusters in the intracellular half of the bilayer and one CS cluster in the extracellular half, as well as more isolated CSs. Considering the additional data available for the MD ensemble mapping and the sampling of novel low-energy CSs that are conserved across multiple conformers, we decided to focus our attention on the MD-based probe dataset.

**Figure 4:**
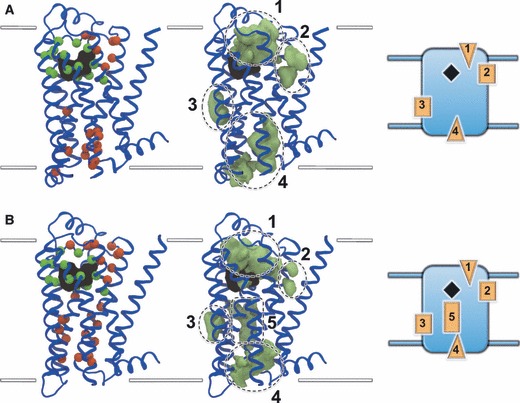
Principal non-orthosteric interaction sites on β_1_AR (A) and β_2_AR (B). Top 40 residues interacting with probe molecules are shown on the left, with orthosteric residues shown as green spheres and non-orthosteric residues shown as red spheres. Probes binding to the non-orthosteric residues are shown in the center as green densities that have been clustered into five key regions, four of which are shared between both receptors and one of which is exclusive to β_2_AR. A schematic depiction of the receptors and their key non-orthosteric sites is shown on the right, with solvent-exposed regions shown as triangles and lipid-exposed regions shown as rectangles. Receptors are shown in blue cartoon representation and depict the experimental conformation. The superimposed co-crystallized ligands are shown in black molecular surface representation. Approximate limits of the bilayer are shown as white lines.

While a visual description of the CSs in the context of the experimental structure is useful for a coarse analysis and illustrative purposes, significant backbone and sidechain movements in the MD structures can take place. To more precisely characterize the dominant non-OSs of interest, we ranked the protein residues in each receptor by the number of non-bonded interactions they make with probe molecules belonging to the CSs (an interaction is counted when a non-hydrogen probe atom is found within a 5 Å radius of a non-hydrogen protein atom). The number of such interactions was summed over all 15 MD structures and then listed as a percentage of all protein–probe interactions for the MD ensemble. This statistic therefore ranks the key binding site residues by their overall performance in the ensemble, as opposed to isolated conformers. After plotting the number of interactions per residue, it was decided to focus on the top 40 probe-interacting residues of each receptor (see Supporting Information). Figure 4 illustrates the distribution of these residues in each receptor and assigns them as either orthosteric or non-orthosteric based on their proximity to the orthosteric ligand in the experimental structures. As expected, a core of orthosteric residues leads the rankings and is then followed by a mixture of orthosteric and non-orthosteric residues. The orthosteric residues line a lower portion of the extracellular mouth, forming a deep cleft. The non-orthosteric residues can be clustered into five distinct groups, along with the probes they bind, as illustrated in Figure 4 and the members of which are listed in Table 1. Both receptors have four non-orthosteric groups in common, with β_2_AR interestingly containing a 5th group not seen in β_1_AR. Sites 1 and 4 are found in the extracellular and intracellular mouths, respectively, while sites 2, 3 and 5 are found at lipid-exposed surfaces. We now explore each of the sites in more detail and in light of available experiment data.

**Table 1:** Key probe-interacting residues forming the 4 non-orthosteric binding sites of β_1_AR and 5 non-orthosteric binding sites of β_2_AR

GPCR	Allosteric site	Key interacting residues (secondary structure)
β_1_AR	1	Gly-115, Ile-118, Val-119 (TM2)
		Cys-216, Asp-217 (ECL2)
		Val-360 (TM7)
	2	Trp-364 (TM7)
	3	Glu-147 (TM3)
	4	Thr-91, Leu-92, Thr-93, Asn-94, Ile-97 (TM2)
		Arg-156 (TM3)
		Tyr-166 (ICL2)
		Pro-285, Pro-286 (ICL3)
		Glu-319, Ala-322, Thr-325, Leu-326 (TM6)
β_2_AR	1	Gly-90, His-93, Ile-94 (TM2)
		Asp-192, Phe-194 (ECL2)
		Trp-99 (ECL1)
		Lys-305, Ile-309 (TM7)
	2	Trp-313 (TM7)
	3	Glu-122, Cys-125 (TM3)
	4	Thr-68, Asn-69 (TM2)
		Arg-131 (TM3)
		Ala-271 (TM6)
	5	Thr-73, Ser-74, Cys-77, Ala-78, Val-81 (TM2)
		Ile-154, Trp-158 (TM4)
		Leu-115 (TM3)

GPCR, G-protein coupled receptor.

### Site 1: extracellular mouth

**Figure 5:**
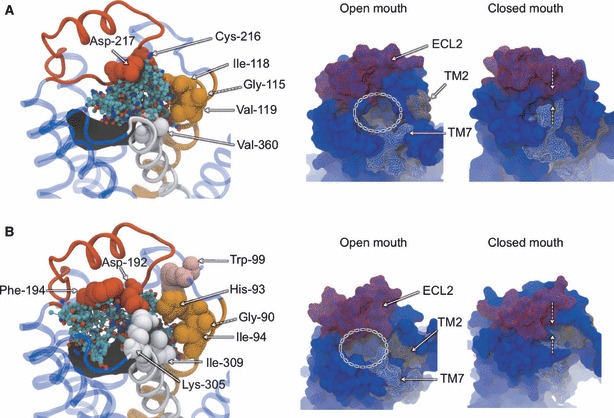
Non-orthosteric Site 1: the extracellular mouth region of β_1_AR (A) and β_2_AR (B). An extracellular view of the mouth region is shown on the left, with bound probe molecules and key interacting residues. Bound probe molecules are shown in ball-and-stick representation and colored by atom type. Key residues are shown in space-filing representation and labeled. Receptors are shown in transparent blue cartoon representation, with TM2 colored orange, TM7 colored white and ECL2 colored red. The experimental conformation of the receptor is shown. The gating motion of the extracellular mouth region seen in the MD simulations is shown on the right, with snapshots of the open and closed conformations. Receptors are shown in blue molecular surface representation, with TM2, TM7 and ECL2 colored orange, white and red, respectively.

Site 1 is found in the upper region of the extracellular mouth, in the solvent-exposed cavity between the OS and the ends of each TM helix (Figure 5). For β_1_AR, the probes tend to bind in the upper-right region of the mouth, toward ECL1, while in β_2_AR probes have a broader distribution and are also found directly above the orthosteric ligand, toward ECL3. On average, FTMAP identifies two CSs in this region for each input MD structure, with a third of the structures reporting the top ranked CS here. The key interacting residues of this site stem from ECL2, TM2 and TM7 (and ECL1 in β_2_AR) and cluster in a corner of the extracellular mouth. β_2_AR contains an additional residue in ECL2 (Phe-194) that is largely responsible for binding probes not seen in the same region in β_1_AR. The locations of the residues are well shared between the receptors, although it is clear that there is greater sequence diversity here, compared to the OS, which makes it an appealing target in terms of subtype selectivity.

The extracellular mouth of GPCRs is probably the most frequently studied region in biochemical work investigating allosteric ligands, so it was encouraging to see such a rich source of CSs found here. Studies on muscarinic receptors have provided evidence for one or more allosteric sites in the extracellular entrance, in a region above and distinct from the deeper OS, consistent with Site 1 found here (31,77). More specifically, residues from ECL2 and TM7, as seen in Site 1, have been shown to interact with allosteric ligands. In ECL2, acidic residues and a downstream tyrosine residue have been proposed to bind allosteric compounds in muscarinic receptors [see (26)], which may correspond to the aspartate residues found in both βARs and the downstream phenylalanine residue found specifically in β_2_AR. Various residues at the top of TM7 and beginning of ECL3 have been implicated in allosteric binding roles in muscarinic receptors [see (26)], including a lysine residue which is also seen in the β_2_AR. While comparable experimental data for βARs is lacking, these correlations suggest they may possess an analogous allosteric site to the muscarinic receptors in this extracellular mouth region.

An important question is how compounds binding in the entrance to the OS exert their allosteric effect. Muscarinic allosteric ligands have been shown to act in a modulatory fashion, by inhibiting either the dissociation or association of orthosteric ligands (i.e. prohibiting their exit or entry to/from the extracellular medium) (78). They have therefore been conceptualized as ‘plugs’, which seal the entrance to the OS and either lock a bound orthosteric ligand in place or prevent its ingress, with functional consequences. Experimental work by Avlani and co-workers on the M_2_ muscarinic receptor has demonstrated that despite the presence of stabilizing disulfide bridges, ECL2 has the flexibility to modulate the accessibility of the OS, thus acting as a ‘gatekeeper’ (79). It was proposed that the open state of the mouth would allow entrance of the orthosteric ligand, followed by a closure that would increase the number of receptor–ligand interactions. The results of our MD simulations suggest that this gating feature is also shared by βARs as the extracellular entrance is capable of motions that completely seal off the water-filled OS. Using principal component analysis, we observed that the dominant mode of global receptor motion in both MD trajectories involved an opening/closing event at the extracellular end of the receptors that was not foreseen from the experimental structures alone. Figure 5 shows snapshots of the two receptors that represent the most open and most closed states, in a movement that is characterized by the movement of ECL2 toward and away from the top of TM7. This movement is consistent with the engineered closed state of the M_2_ muscarinic receptor, whereby a disulfide bridge was introduced between sites corresponding to a valine residue in ECL2 and an asparagine residue at the top of TM7 (79). Avlani and co-workers proposed that allosteric ligands binding in the vicinity of this gate region would therefore serve to either (i) bind to and stabilize the closed form of the gate or (ii) bind to the open form and therefore mimic the closed state of the gate. Our results suggest that the former is more likely in the case of βARs, as all probe-binding sites identified by FTMAP would be concealed in the closed state and therefore inaccessible. A bound allosteric ligand would therefore bridge the opposing components of the gate and block the orthosteric ligand pathway. Further evidence for this blocking model comes from our related work on the binding of auto-antibodies to an antigenic site on ECL2 of the β_1_AR in Chagas’ disease (to be published elsewhere). Auto-antibody fragments have been experimentally shown to block the accessibility of the OS in β_1_AR (80), and our protein–protein docking results suggest that antibody loops partially enter the extracellular mouth and cover the entrance. In the case of β_2_AR, a recent NMR study has supported the dynamic nature of the extracellular mouth, whereby the formation of a salt bridge between Lys-305 of TM7 and Asp-192 of ECL2 is shown to reflect the activation state of the receptor (81). Interestingly, these residues are both identified in our study, and it was proposed that allosteric ligands binding in this vicinity might also be able to influence the state of the receptor by themselves (i.e. independently of the orthosteric ligand). Finally, a third potential class of allosteric ligand has been proposed in this region, as it annexes the OS: so-called ‘bitopic’ or ‘dualsteric’ ligands (78,82). Such ligands represent the fusion of an orthosteric and allosteric ligand, whereby the allosteric component confers subtype specificity and the orthosteric component exerts functional effects. Our results suggest that the rational design of such ligands is feasible in the case of βARs as there is sufficient spatial proximity between the OS and the bound probe molecules for a small molecule to span both locations. Indeed, there appears to be a degree of overlap between the orthosteric and non-orthosteric probe molecules, as has been noted for certain allosteric modulators (31). The MD structures also demonstrate that the extracellular mouth has the capacity to accommodate both elements of bitopic ligands simultaneously, as probes are typically bound at both sites for the same input structure.

### Site 2: TM1-TM7 cavity

**Figure 6:**
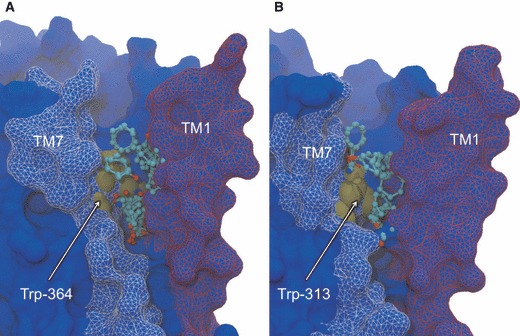
Non-orthosteric Site 2: the TM1–TM7 cavity of β_1_AR (A) and β_2_AR (B). Side view of the cavity taken in the plane of the bilayer, depicting bound probe molecules and the key interacting tryptophan residue. Bound probe molecules are shown in ball-and-stick representation and colored by atom type. Tryptophan residue is shown in brown space-filling representation and labeled. Receptors are shown in blue molecular surface representation, with TM1 colored red and TM7 colored white. The experimental conformation of the receptor is shown.

Site 2 is formed by a ‘U’-shaped cavity that lies in between the extracellular ends of TM1 and TM7, in the upper leaflet of the bilayer (Figure 6). This space is segregated from the extracellular mouth region by TM2 and thus lies at a protein–lipid interfacial region. During both MD trajectories, this void is largely populated by the fatty acyl chains of POPC phospholipid molecules, confirming the hydrophobic environment of the pocket. This observation underscores the value of a short CG simulation to determine the initial positioning of phospholipids, particularly in the annular shell coating the receptor. FTMAP detects a single CS in this pocket in seven of the β_1_AR MD structures and five of the β_2_AR MD structures. While the CS is found in the experimental β_1_AR structure, it is not seen in the experimental β_2_AR structure, only appearing through MD-induced relaxations. This transient character contrasts with the solvent-exposed Site 1 and suggests this pocket may form less frequently and may be associated with particular states of the GPCR. The key interacting residue of Site 2 is a tryptophan in TM7 that is conserved in both receptors (Trp-364 in β_1_AR and Trp-313 in β_2_AR). However, the peripheral sidechains lining the site show differences that could be exploited in the design of subtype-selective ligands. While experimental data supporting a role for allosteric ligand binding in this pocket is lacking, one might predict the conformational consequences of small molecules ‘wedging’ this cavity open. For example, TM7 contributes residues to the orthosteric binding site, and therefore allosteric ligands may stabilize conformations that favor orthosteric ligand binding or release. The intracellular end of TM7 also contributes the ‘NPXXY’ motif, which is thought to be involved in G-protein activation and might be constrained by a less mobile TM7. Another possibility is that allosteric ligands at this site may stabilize the open state of the extracellular mouth, as based on the motions depicted in Figure 5, the movement of TM7 toward ECL2 may be hindered. We note that the experimental structures used in this study do not contain an N-terminal fragment of 55 (β_1_AR) and 28 (β_2_AR) residues that contained disordered and deleted regions in the crystallization constructs. The fact that this sequence annexes TM1 means we cannot exclude the possibility that its omission affects the dynamics of this binding pocket.

### Site 3: TM4–TM3–TM5 junction

**Figure 7:**
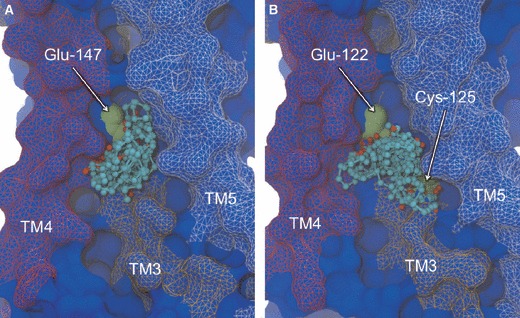
Non-orthosteric Site 3: the TM4-TM3-TM5 junction of β_1_AR (A) and β_2_AR (B). Side view of the cavity taken in the plane of the bilayer, depicting bound probe molecules and the key interacting residues. Bound probe molecules are shown in ball-and-stick representation and colored by atom type. Key residues are shown in green space-filling representation and labeled. Receptors are shown in blue molecular surface representation, with TM4 colored red, TM3 colored orange and TM5 colored white. The experimental conformation of the receptor is shown.

Site 3 is formed by a cavity at the intersection of TM3, TM4 and TM5, in the lower leaflet of the bilayer (Figure 7). This site is found on the exterior surface of the TM core and is therefore also located at the protein–lipid interface. Similarly to Site 2, the pocket is occupied by the fatty acyl chains of phospholipid molecules and therefore represents another opportunity for potential hydrophobic drugs to enter from the membrane. Single CSs are detected in 11 and 10 of the β_1_AR and β_2_AR MD structures, respectively, again suggesting a transient nature whereby the chemistry and geometry of the pocket change with protein dynamics. In the case of β_2_AR, four of the structures also yield a second CS, suggesting the pocket may be slightly larger for this receptor. Interestingly, this site is not exposed by FTMAP in either of the experimental structures, further supporting the role of incorporating target flexibility in the detection of putative drug-binding sites. Conserved at Site 3 in both receptors is a glutamate residue (Glu-147 in β_1_AR and Glu-122 in β_2_AR) that interacts through backbone and methylene atoms, along with further notable interactions from Cys-125 in β_2_AR (also conserved in β_1_AR). As noted in Site 2, non-identical residues are found at other locations in the pocket, which could support receptor selectivity. Occurring at the junction between three of the seven TM helices, it is likely that ligands binding in, and therefore occluding, this cavity will have a strong influence on the conformational flexibility of the receptor, potentially stabilizing certain activation states. In particular, TM3 contains residues upstream of the site that interact with orthosteric ligands and a downstream residue engaged in the ‘ionic lock’, proposed to be an important conformational switch in GPCR activation. Thus, allosteric ligands at this site may have the capacity to either modulate orthosteric ligand binding and/or influence GPCR activation autonomously, by constraining key TM helices. Interestingly, a recent experimental study of β_1_AR and β_2_AR reported the substitution of bulky hydrophobic residues for Glu-147 and Glu-122 in Site 3 (83), with the aim of stabilizing GPCRs in structural biology applications. The mutations were found to substantially increase the conformational stability of these GPCRs, and it was proposed that this was induced by reducing the flexibility of TM5, which contains a proline-induced break which lines the binding pocket. The appearance of favorable probe-binding sites in the vicinity of these glutamate residues in our work would therefore appear to mimic such mutagenesis experiments and suggest small molecules directed to this site may have important functional consequences. Furthermore, it provides another potential role for allosteric ligands in the facilitation of GPCR structural biology experiments.

### Site 4: intracellular mouth

**Figure 8:**
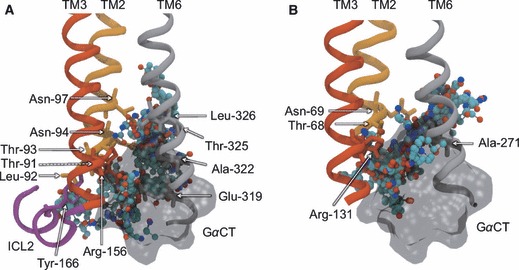
Non-orthosteric Site 4: the intracellular mouth region of β_1_AR (A) and β_2_AR (B). A view of the mouth region taken in the plane of the bilayer, depicting bound probe molecules and the key interacting residues. Bound probe molecules are shown in ball-and-stick representation and colored by atom type. Key residues are shown in stick representation and labeled. Key TM helices are shown in cartoon representation, with TM2 colored orange, TM3 colored red, TM6 colored gray and ICL2 colored purple. The experimental conformation of the receptor is shown, with other structures obscured for clarity. The superimposed GαCT fragment is shown in transparent black molecular surface representation.

Site 4 is found in the solvent-exposed cavity formed at the intracellular entrance to the TM core (Figure 8). This pocket is smaller than the extracellular opening, with its size and accessibility modulated by movements of the TM helices and the large ICL3, respectively. Outward movements of the TM helices in the MD simulations allow probes to permeate deeper into the core, particularly so in the vicinity of TM6. FTMAP detects CSs in this region for almost all structures, typically with 1–3 clusters reported, suggesting it can accommodate relatively large molecules. In both receptors, key interacting residues are located on TM2, TM3 and TM6, with an additional contribution from ICL2 and ICL3 in β_1_AR. The abundance of probe-binding sites is perhaps not surprising given this is a known protein–protein interaction site between GPCRs and their cognate G-proteins. The recent crystal structure of bovine opsin complexed with a synthetic peptide corresponding to a C-terminal portion of a G-protein α subunit (GαCT) confirms this (19). The surface of the superimposed GαCT fragment accommodates the majority of the probes found in the mapping of this site, suggesting most of them have overlapping binding sites with G-proteins (Figure 8). However, the aforementioned deeper probes do appear to bind in regions distinct from those occupied by the GαCT, along with a cluster of probes in the vicinity of ICL2 in β_1_AR. It is therefore unclear if potential compounds designed to interact with this site would compete with native GPCR:G–protein interactions or bind in addition to the G-protein. In the former case, competitive allosteric ligands could obviously be used to attenuate GPCR signalling, by blocking the G-protein interaction site. In the latter case, as it is known that the opening of the intracellular mouth is required to bind the G-protein in the case of opsin, one could imagine an allosteric modulator that binds deep in the pocket and stabilizes this open/active state to enhance signalling. Interestingly, zinc ions have been shown to have an allosteric impact on β_2_AR, with mutagenesis experiments pinpointing their interaction site to a cluster of residues at the base of TM5 and TM6, at the intracellular mouth (84). It has been shown that zinc binding at this site facilitates the binding of the distant orthosteric ligand, and it has been proposed that the ions bridge TM5 and TM6 and stabilize an activated state of the GPCR. While this site does not appear in our mapping of the intracellular mouth, we note that key interactions are instead made between probe molecules and elements of another key conformational switch – the ionic lock (Arg-156 and Glu-319 in β_1_AR and Arg-131 in β_2_AR). The separation of this salt bridge is thought to be a hallmark of activated GPCR states and thus small molecules binding in between them may stabilize these states, preventing reformation of the lock.

### Site 5: cholesterol-binding site

**Figure 9:**
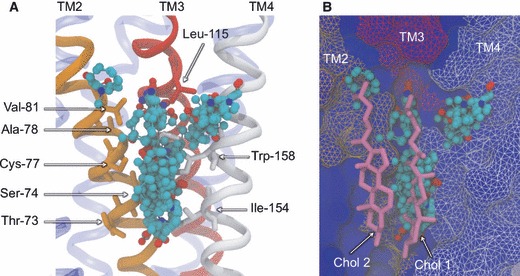
Non-orthosteric Site 5: the cholesterol-binding site of β_2_AR. A view of the cholesterol-binding region taken in the plane of the bilayer, depicting bound probe molecules and the key interacting residues. Bound probe molecules are shown in ball-and-stick representation and colored by atom type. Key residues are shown in stick representation and labeled. Key TM helices are shown in cartoon representation (A), with TM2 colored orange, TM3 colored red and TM4 colored white. β_2_AR is also shown in blue molecular surface representation (B), along with the two superimposed cholesterol structures, shown in pink stick representation, to show overlap between probe molecules and cholesterol 1. The experimental conformation of β_2_AR is shown.

Site 5 is found at the intersection of TM2, TM3 and TM4, in the lower leaflet of the lipid bilayer (Figure 9). This site represents the third lipid-exposed groove discovered and is also lined with the fatty acyl tail of POPC lipids in the MD simulations. Site 5 is unique in that it is the only binding pocket identified exclusively in one of the receptor types –β_2_AR. For this structure, between 1 and 3 CSs are detected in 10 of the 15 MD structures, with the two most populated MD representative structures yielding the largest CS in this area. Consequently, 8 of the top 40 probe-binding residues from the β_2_AR analysis stem from the neighbouring TMs. In contrast, single CSs are identified in only two of the 15 β_1_AR MD structures, with no residues identified in the top 40. For both experimental structures, no probe molecules were found at this site, requiring MD simulation to become exposed. The clear difference in the predicted probe-binding capacity of this region poses important possibilities for subtype-selective drugs, if ligand interactions at this site have functional consequences. Cholesterol has been known to modulate the function of several GPCRs (85), and a new β_2_AR crystal structure has recently clarified the significance of co-crystallized cholesterol molecules that are seen to bind in the lower half of the receptor (86). It was shown that there is a pair of cholesterol molecules that binds specifically to a single site between TMs 1–4 and does not appear to be a crystal packing artifact. Furthermore, it was reported that a cholesterol analog increased the conformational stability of the receptor and that cholesterol can modulate the orthosteric ligand-binding properties, suggestive of an allosteric effect. Figure 9B shows the superposition of the pair of co-crystallized cholesterol molecules with Site 5 of β_2_AR and the bound probe molecules from our study. The majority of the probes overlap well with cholesterol site 1 (which produces more protein interactions than site 2), suggesting small molecules directed to this site may be able to mimic the allosteric effects of cholesterol. Such compounds would effectively fill the void between the three TM helices, in a manner similar to that shown for Site 3 in Figure 7, constraining the conformational flexibility of the receptor and potentially stabilizing certain conformers. Indeed, Hanson and co-workers (86) have proposed that the cholesterol-binding site represents a potential therapeutic target. Interestingly, it has been shown that the lipophilic βAR agonist salmeterol interacts with β_2_AR at *both* the OS and an ‘exosite’ involving residues 149–158 of TM4 (87). It is thought that this lipid-exposed secondary site, which overlaps with residues identified in our study, allows the drug to become ‘anchored’ to the receptor and is responsible for its desirable prolonged action. Site 5 may therefore form part of the salmeterol exosite, providing an interaction site for the lipophilic tail of the drug. Importantly, this allosteric site is not found in β_1_AR, supporting our observations and demonstrating this site can confer subtype specificity. Concerning the disparity between the predicted druggability of this site in both receptors, we have compared the amino acids lining the region to identify the chemical basis. Based on the residues interacting with probes in β_2_AR, the three substitutions are Cys-77->Ser-102 and Thr-73->Met-98 in TM2 and Ile-154->Val-179 in TM4 (β_2_AR->β_1_AR). Based on the strict cholesterol consensus motif (strict-CCM) proposed as a cholesterol-binding signature (86), the important substitution is Tyr-70->Leu-95 (β_2_AR->β_1_AR), which is not a strong probe-binding site in our work. We speculate that the lack of probe binding at this site in β_1_AR might reflect a similar absence of cholesterol binding and that the CCM may need to be expanded to embrace other residues, particularly in TM2.

## Conclusions

The allosteric modulation of GPCR activity is the focus of a growing branch of drug discovery, searching for novel therapeutic agents to control the numerous pathologies they play a role in. Among a range of advantages over their orthosteric counterparts, allosteric ligands offer the prospect of highly specific targeting, by binding to less conserved regions of the receptor surface. Despite the development of experimental screening methods for allosteric GPCR lead discovery, *in silico* structure-based approaches are lacking, largely because of the renowned problems associated with GPCR crystallization. In the absence of experimental structures of GPCRs complexed with allosteric ligands, there is a clear window of opportunity for predictive computational approaches using recent unbound structures. In this work, we report the application of a fragment-based algorithm, FTMAP, to map the surface of the human β_1_AR and β_2_AR GPCR structures for druggable sites distinct from the OS. To incorporate the flexibility of the receptors, we have mapped a series of 15 diverse structures taken from a series of MD simulations of each receptor in a phospholipid bilayer.

By focusing on the key interacting protein residues, we have defined a set of five putative allosteric binding sites, four of which are shared between receptor types and one of which is unique to β_2_AR, which we have interpreted with corroborating experimental evidence. Sites 1 and 4 are found in the solvent-exposed extracellular and intracellular mouths, with Site 1 representing a well-known region of allosteric ligand-binding activity in related GPCRs. Key gating motions from MD simulations suggest that allosteric ligands binding at the extracellular mouth may block the entrance or exit of orthosteric ligands by bridging opposing structures at the entrance. The possible function of allosteric ligands at Site 4 is less clear, as this is an interaction site for G-proteins; however, we speculate they may be capable of stabilizing the open form of the cavity and influencing the conformation of the ionic lock. Sites 2, 3 and 5 represent pockets formed at the protein–lipid interface, in the hydrophobic core of the lipid bilayer. Occupying the junctions of TM helices, it is likely that compounds filling these locations would increase inter-helical packing interactions and thus restrict conformational flexibility. This effect is supported by experimental evidence at Sites 3 and 5, where occlusion of the pockets has been shown to increase stability of the receptors and may stabilize distinct states with desirable therapeutic effects. Site 5 is of particular interest as significant probe-binding events are only seen in β_2_AR, suggesting it may be an excellent target for β_2_AR-selective therapies. While structural evidence of protein–drug interactions in the hydrophobic core of the membrane is lacking, a number of lipophilic drugs have demonstrated strong membrane partitioning coefficients and have been proposed to access their membrane-associated receptors from the lipid phase rather than the aqueous phase (88).

From a methodological point of view, the use of MD simulations to model flexibility of the receptors allowed FTMAP to detect some sites not apparent in the static experimental structures and for us to observe the transient nature of some pockets. Sampling different receptor conformations is especially appealing for such flexible proteins as GPCRs, as even subtle rearrangements may expose or conceal key ‘cryptic’ binding sites. Given the broad distribution of the representative MD structures used in this study (see Supporting Information), we conclude that a multi-copy simulation approach of this timescale is successful in generating enhanced conformational diversity. However, despite recent advances in the evolution of computer hardware, conventional MD simulations still suffer from incomplete conformation sampling for systems of this size. It seems reasonable to predict that a more extensive exploration of the conformational landscape could lead to the identification of further druggable binding sites. It is therefore tempting to apply new computational methods in the generation of more diverse structural ensembles. Such methods include accelerated MD (89), replica exchange (90) and conformational flooding (91) and may expose sites that are formed in regions of the energy landscape distant from the experimentally captured conformation.

Having identified a series of potential allosteric binding sites, this work will serve as a springboard for structure and fragment-based lead identification methods. An obvious starting point is in the virtual screening of *existing* drug-like compound libraries for potential high-affinity ligands at each pocket, which can then be assayed for binding and allosteric activity. An alternative approach involves the design and synthesis of *novel* compounds using the poses of docked probe molecules from our analysis with fragment-based techniques (92). Probes can be grown into high-affinity small molecules that interact with further protein residues (fragment evolution). Also, when multiple probes are bound simultaneously, they can be fused to form a single molecule (fragment linking). This approach could also be used to form proposed bitopic compounds in the extracellular mouth of the receptors, by joining orthosteric compounds to putative allosteric probes (82). While the GPCRs featured in this study are logical targets to begin such screening studies and are of considerable therapeutic interest in their own right, it is likely that additional druggable sites are present on related GPCRs, which may be amenable to homology modeling approaches for a similar analysis.

## Supporting Information

**Figure S1.** Conformational drift and extractionof representative structures from the MD simulations of β_1_AR (A) and β_2_AR (B).

**Figure S2.** Non-bonded interactions betweenresidues of β_1_AR (A) and β_2_AR (B) and bound probe molecules from the mapping of the MD ensemble.

**Table S1.** List of 40 residues engaged in thestrongest protein–probe interactions for β_1_AR and β_2_AR, from the mapping of the MD ensemble.
