# Supplementary figures and images for: Mapping the Druggable Allosteric Space of G-Protein Coupled Receptors: a Fragment-Based Molecular Dynamics Approach
Source: Chem Biol Drug Des. 2010 Sep;76(3):201–17. doi: 10.1111/j.1747-0285.2010.01012.x (PMC2918726; doi:10.1111/j.1747-0285.2010.01012.x)

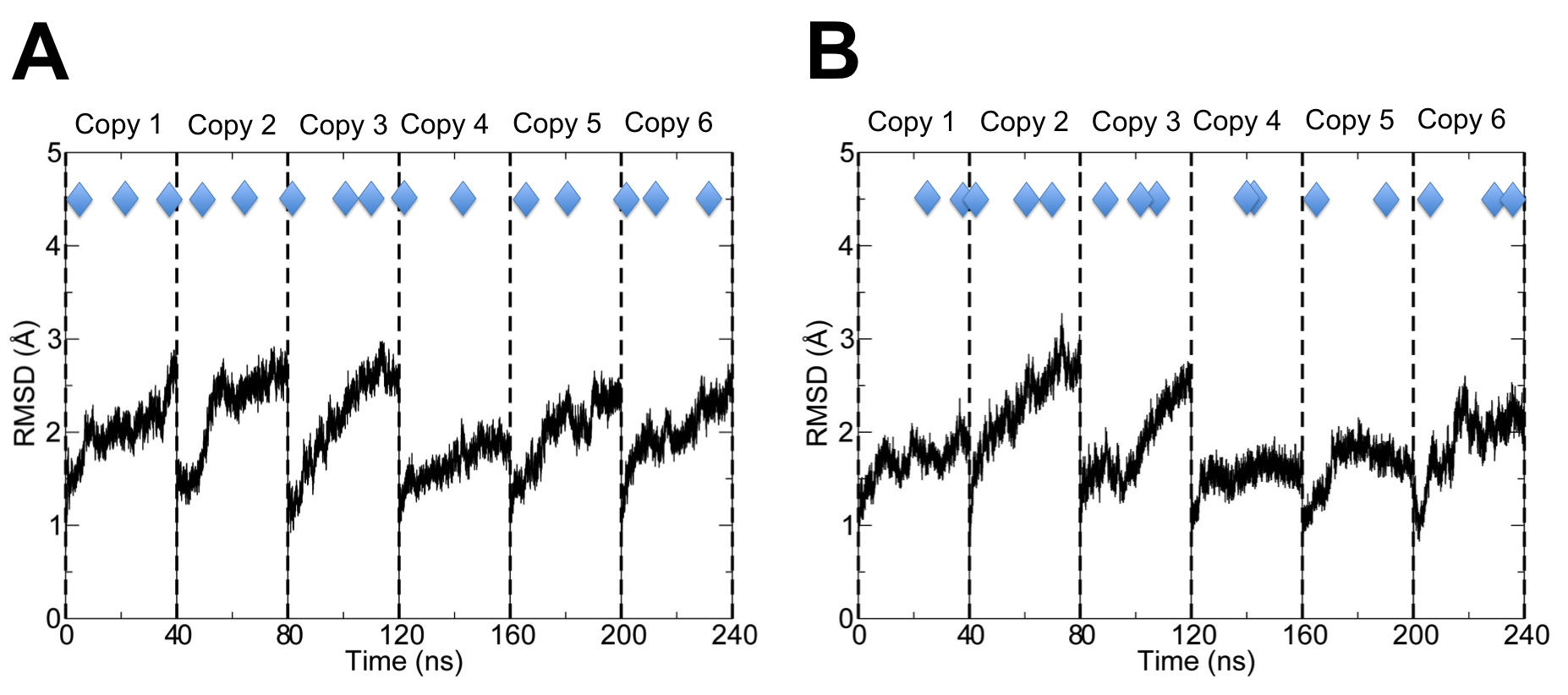

Supplement: Supplementary file 1 [file cbdd0076-0201-SD2.tif]

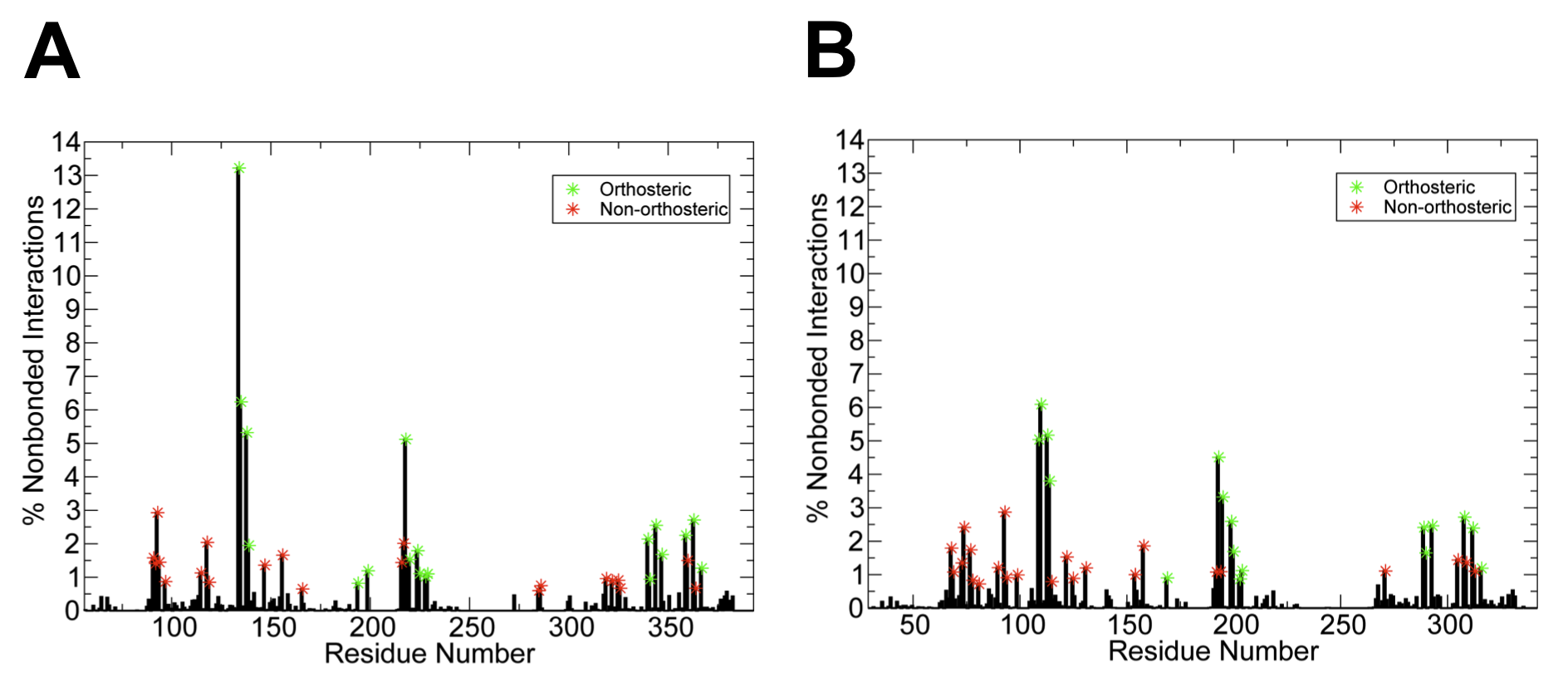

Supplement: Supplementary file 2 [file cbdd0076-0201-SD3.tif]
