# Supplementary material for: Mapping the Druggable Allosteric Space of G-Protein Coupled Receptors: a Fragment-Based Molecular Dynamics Approach
Source: Chem Biol Drug Des. 2010 Sep;76(3):201–17. doi: 10.1111/j.1747-0285.2010.01012.x (PMC2918726; doi:10.1111/j.1747-0285.2010.01012.x)
Supplement: Supplementary file 3 [file cbdd0076-0201-SD1.doc]

| **β_1_AR** | | | | | **β_2_AR** | | | | |
| --- | --- | --- | --- | --- | --- | --- | --- | --- | --- |
| Rank | Residue | Loc. | OS? | %Int. | Rank | Residue | Loc. | OS? | %Int. |
| 1 | TRP-134 | TM3 | * | 13.22 | 1 | THR-110 | TM3 | * | 6.09 |
| 2 | THR-135 | TM3 | * | 6.24 | 2 | ASP-113 | TM3 | * | 5.17 |
| 3 | ASP-138 | TM3 | * | 5.32 | 3 | TRP-109 | TM3 | * | 5.04 |
| 4 | PHE-218 | ECL2 | * | 5.12 | 4 | PHE-193 | ECL2 | * | 4.51 |
| 5 | THR-93 | TM2 |  | 2.93 | 5 | VAL-114 | TM3 | * | 3.80 |
| 6 | ASN-363 | TM7 | * | 2.71 | 6 | THR-195 | ECL2 | * | 3.32 |
| 7 | ASN-344 | TM6 | * | 2.55 | 7 | HIS-93 | TM2 |  | 2.87 |
| 8 | PHE-359 | TM7 | * | 2.25 | 8 | TYR-308 | TM7 | * | 2.72 |
| 9 | PHE-340 | TM6 | * | 2.14 | 9 | TYR-199 | TM5 | * | 2.59 |
| 10 | ILE-118 | TM2 |  | 2.04 | 10 | ASN-293 | TM6 | * | 2.46 |
| 11 | ASP-217 | ECL2 |  | 2.01 | 11 | SER-74 | TM2 |  | 2.41 |
| 12 | VAL-139 | TM3 | * | 1.95 | 12 | PHE-289 | TM6 | * | 2.41 |
| 13 | TYR-224 | TM5 | * | 1.79 | 13 | ASN-312 | TM7 | * | 2.39 |
| 14 | LYS-347 | TM6 | * | 1.68 | 14 | TRP-158 | TM4 |  | 1.86 |
| 15 | ARG-156 | TM3 |  | 1.66 | 15 | THR-68 | TM2 |  | 1.79 |
| 16 | THR-91 | ICL1 |  | 1.58 | 16 | CYS-77 | TM2 |  | 1.74 |
| 17 | THR-220 | ECL2 | * | 1.53 | 17 | ALA-200 | TM5 | * | 1.69 |
| 18 | VAL-360 | TM7 |  | 1.50 | 18 | PHE-290 | TM6 | * | 1.65 |
| 19 | ASN-94 | TM2 |  | 1.45 | 19 | GLU-122 | TM3 |  | 1.53 |
| 20 | CYS-216 | ECL2 |  | 1.44 | 20 | LYS-305 | TM7 |  | 1.44 |
| 21 | LEU-92 | TM2 |  | 1.41 | 21 | ILE-309 | TM7 |  | 1.36 |
| 22 | GLU-147 | TM3 |  | 1.36 | 22 | THR-73 | TM2 |  | 1.35 |
| 23 | TYR-367 | TM7 | * | 1.27 | 23 | GLY-90 | TM2 |  | 1.21 |
| 24 | TRP-199 | ECL2 | * | 1.19 | 24 | ARG-131 | TM3 |  | 1.20 |
| 25 | GLY-115 | TM2 |  | 1.13 | 25 | TYR-316 | TM7 | * | 1.19 |
| 26 | ALA-225 | TM5 | * | 1.11 | 26 | SER-204 | TM5 | * | 1.12 |
| 27 | SER-229 | TM5 | * | 1.10 | 27 | ALA-271 | TM6 |  | 1.11 |
| 28 | SER-228 | TM5 | * | 1.02 | 28 | TRP-313 | TM7 |  | 1.10 |
| 29 | GLU-319 | TM6 |  | 0.96 | 29 | PHE-194 | ECL2 |  | 1.09 |
| 30 | PHE-341 | TM6 | * | 0.94 | 30 | ASN-69 | TM2 |  | 1.08 |
| 31 | THR-325 | TM6 |  | 0.91 | 31 | ASP-192 | ECL2 |  | 1.08 |
| 32 | ALA-322 | TM6 |  | 0.89 | 32 | ILE-154 | TM4 |  | 1.00 |
| 33 | ILE-97 | TM2 |  | 0.87 | 33 | TRP-99 | ECL1 |  | 0.99 |
| 34 | VAL-119 | TM2 |  | 0.85 | 34 | ILE-94 | TM2 |  | 0.91 |
| 35 | ILE-194 | TM4 | * | 0.82 | 35 | ILE-169 | TM4 | * | 0.90 |
| 36 | PRO-286 | ICL3 |  | 0.75 | 36 | CYS-125 | TM3 |  | 0.89 |
| 37 | TRP-364 | TM7 |  | 0.67 | 37 | SER-203 | TM5 | * | 0.87 |
| 38 | LEU-326 | TM6 |  | 0.67 | 38 | ALA-78 | TM2 |  | 0.83 |
| 39 | TYR-166 | ICL2 |  | 0.65 | 39 | LEU-115 | TM3 |  | 0.79 |
| 40 | PRO-285 | ICL3 |  | 0.60 | 40 | VAL-81 | TM2 |  | 0.72 |
